# Supplementary material for: Discovery of New Trichophyton Members, T. persicum and T. spiraliforme spp. nov., as a Cause of Highly Inflammatory Tinea Cases in Iran and Czechia
Source: Microbiol Spectr. 2021 Sep 1;9(2):e00284-21. doi: 10.1128/Spectrum.00284-21 (PMC8557871; doi:10.1128/Spectrum.00284-21)
Supplement: SUPPLEMENTAL FILE 1 — Supplemental material. Download SPECTRUM00284-21_Supp_1_seq14.pdf, PDF file, 0.2 MB. [file spectrum00284-21_supp_1_seq14.pdf]

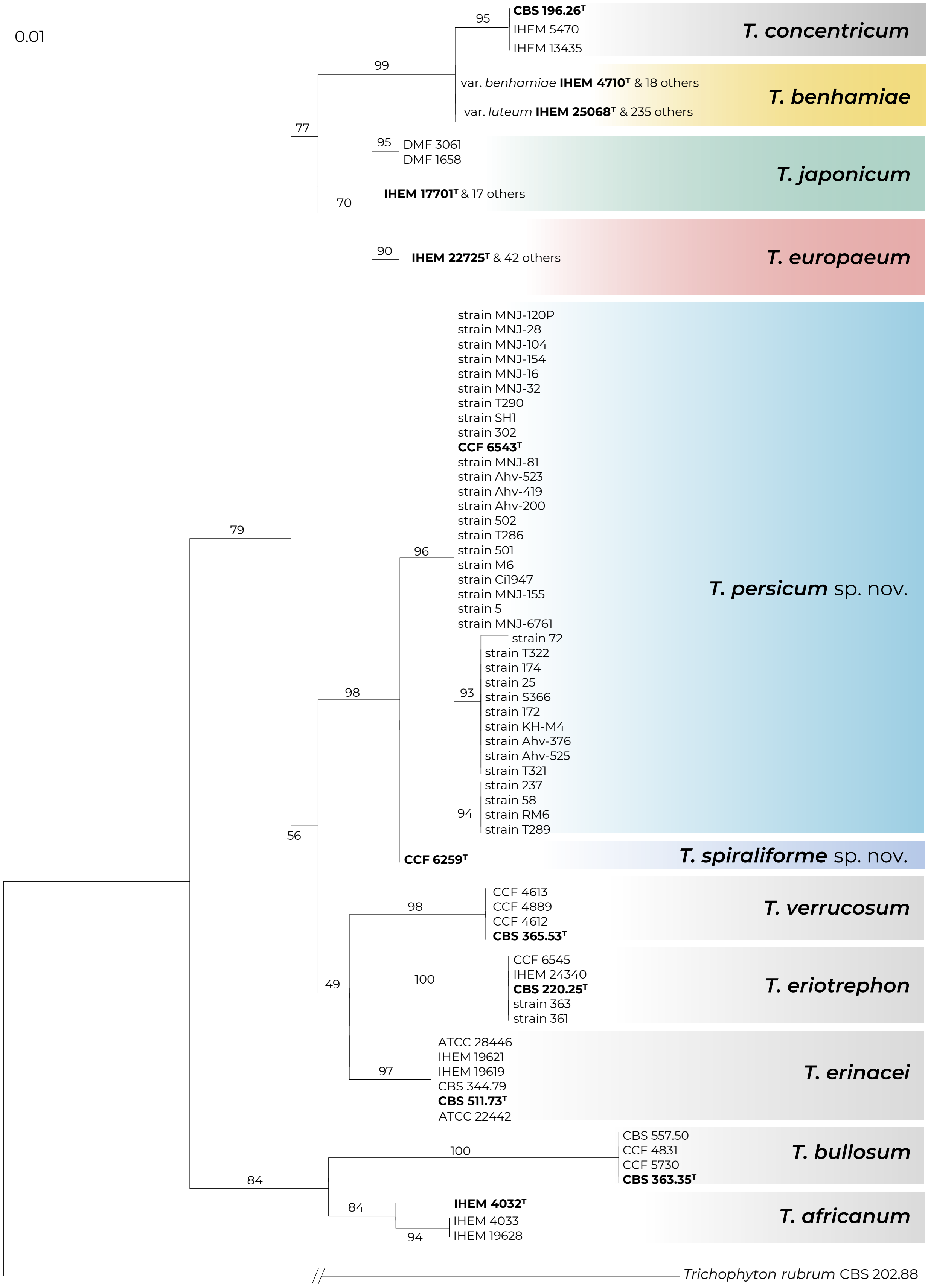

Supplementary Figure S1. A best scoring Maximum Likelihood tree based on ITS rDNA region showing relationships of *Trichophyton spiraliforme* sp. nov. and *Trichophyton persicum* sp. nov. to other dermatophytes belonging to the *T. benhamiae* complex. Ex-type isolates are designated by a superscript T. *Trichophyton rubrum* CBS 202.88 was used as the outgroup.

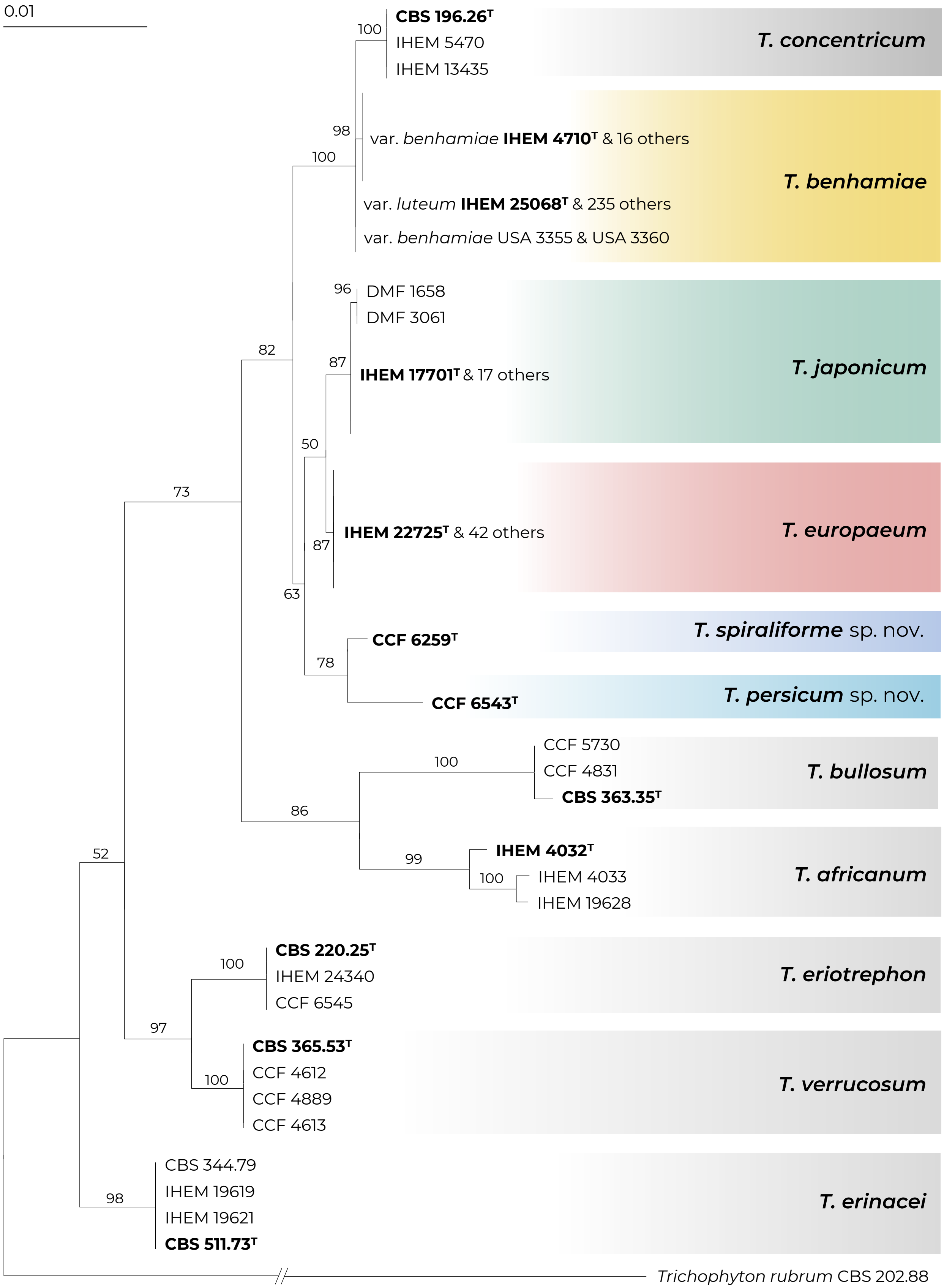

Supplementary Figure S1. A best scoring Maximum Likelihood tree that includes only strains with complete sequence data available for four loci (*gapdh*, *tubb*, ITS rDNA and *tef1-α*). The tree shows the relationships of *Trichophyton spiraliforme* sp. nov. and *Trichophyton persicum* sp. nov. to other dermatophytes belonging to the *T. benhamiae* complex. Ex-type isolates are designated by a superscript T. *Trichophyton rubrum* CBS 202.88 was used as the outgroup.

TABLE S1. Species-specific conserved substitutions in the ITS rDNA region of *Trichophyton benhamiae* clade species		
**Compared taxa**	**Number of substitutions**	**Position of conserved substitution / indel** [1]
T. benhamiae × *T. concentricum*	2	80 (C→T), 526 (T→C)
T. benhamiae × *T. europaeum*	6	41 (A→G), 43 (T→C), 54 (A→G), 266 (T→C), 471 (A→G), 475 (T→C); indel 609
T. benhamiae × *T. japonicum*	7	41 (A→G), 43 (T→C), 54 (A→G), 168 (T→C), 266 (T→C), 471 (A→G), 475 (T→C); indel 609
T. benhamiae × *T. persicum*	12	41 (A→G), 168 (T→C), 230 (G→A), 266 (T→C), 437 (C→T), 456 (A→G), 471 (A→G), 475 (T→C), 476 (C→T), 550 (G→A), 562 (C→T), 564 (C→T); indel 213
T. benhamiae × *T. spiraliforme*	10	41 (A→G), 168 (T→C), 230 (G→A), 266 (T→C), 471 (A→G), 475 (T→C), 476 (C→T), 550 (G→A), 562 (C→T), 564 (C→T); indel 213
T. concentricum × *T. persicum*	14	41 (A→G), 80 (T→C), 168 (T→C), 230 (G→A), 266 (T→C), 437 (C→T), 456 (A→G), 471 (A→G), 475 (T→C), 476 (C→T), 526 (C→T), 550 (G→A), 562 (C→T), 564 (C→T); indel 213
T. concentricum × *T. europaeum*	8	41 (A→G), 43 (T→C), 54 (A→G), 80 (T→C), 266 (T→C), 471 (A→G), 475 (T→C) & 526 (T→C); indel 609
T. concentricum × *T. japonicum*	9	41 (A→G), 43 (T→C), 54 (A→G), 80 (T→C), 168 (T→C), 266 (T→C), 471 (A→G), 475 (T→C) & 526 (C→T); indel 609
T. concetricum × *T. spiraliforme*	12	41 (A→G), 80 (T→C), 168 (T→C), 230 (G→A), 266 (T→C), 471 (A→G), 475 (T→C), 476 (C→T), 526 (C→T), 550 (G→A), 562 (C→T), 564 (C→T); indel 213
T. europaeum × *T. persicum*	10	43 (C→T), 54 (G→A), 168 (T→C), 230 (G→A), 437 (C→T), 456 (A→G), 476 (C→T), 550 (G→A), 562 (C→T), 564 (C→T); indel 213, indel 609
T. europaeum × *T. spiraliforme*	8	43 (C→T), 54 (G→A), 168 (T→C), 230 (G→A), 476 (C→T), 550 (G→A), 562 (C→T), 564 (C→T); indel 213, indel 609
T. japonicum × *T. persicum*	9	43 (C→T), 54 (G→A), 230 (G→A), 437 (C→T), 456 (A→G), 476 (C→T), 550 (G→A), 562 (C→T), 564 (C→T); indel 213, indel 609
T. japonicum × *T. europaeum*	1	168 (C→T)
T. japonicum × *T. spiraliforme*	7	43 (C→T), 54 (G→A), 230 (G→A), 476 (C→T), 550 (G→A), 562 (C→T), 564 (C→T); indel 213, indel 609
T. persicum × *T. spiraliforme*	2	437 (T→C), 456 (G→A)

[1] positions based on the ITS alignment deposited in the Dryad digital repository
